# Diagnostic and Prognostic Markers in Bladder Cancer

**DOI:** 10.1155/2016/2425091

**Published:** 2016-09-29

**Authors:** Ja Hyeon Ku, Wun-Jae Kim, Seth P. Lerner, Felix Chun, Luis Alex Kluth

**Affiliations:** ^1^Seoul National University Hospital, Seoul, Republic of Korea; ^2^Chungbuk National University, Cheongju, Republic of Korea; ^3^Baylor College of Medicine, Houston, TX, USA; ^4^University Hospital Hamburg-Eppendorf, Hamburg, Germany; ^5^University Medical Center Hamburg-Eppendorf, Hamburg, Germany

Bladder cancer has the highest recurrence rate of any solid tumor. Due to the need for life-long surveillance, the per-patient cost of managing bladder cancer is among the highest for any cancer. There has been growing activity in the development of novel markers for bladder cancer detection; the ultimate goal of these novel markers would be to replace and/or complement cystoscopy as a sensitive and noninvasive surveillance tool. Besides, with recent advances in techniques, an extremely large number of new prognostic markers have been identified. New biomarkers that are predictive of outcomes would help clinicians provide the risk-stratification of patients and serve as prognostic indicators for individual patients. Continuing identification of new markers in bladder cancer will enhance future diagnostic and therapeutic approaches to bladder cancer.

We invited investigators to contribute articles that will stimulate continuing efforts to leverage biomarkers to improve diagnostic accuracy, discover the molecular pathophysiology underlying bladder cancer, develop strategies to treat these conditions, and evaluate the prognosis. The purpose of this special issue is to give a comprehensive overview of the current state of knowledge regarding diagnostic and prognostic markers in bladder cancer.

Several studies have indicated that steroid hormones and their receptor signals, especially androgens/estrogens and androgen/estrogen receptors, in bladder cancer, have critical roles in tumorigenesis and tumor progression. In one review, H. Ide and H. Miyamoto summarized that steroid hormone receptors and related signals can serve as biomarkers of urothelial tumors, especially their prognosticators.

In “Association of Cytokeratin and Vimentin Protein in the Genesis of Transitional Cell Carcinoma of Urinary Bladder Patients,” A. H. Rahmani et al. performed immunohistochemistry and investigated cytokeratin and vimentin in urothelial cancer cases and inflammatory lesions. They concluded that both markers cytokeratin and vimentin will be helpful markers in the early diagnosis of urothelial carcinoma.

M. S. Wettstein et al. evaluated CD73 expression immunohistochemically in 174 patients with a primary urothelial carcinoma. They found that high CD73 expression was associated with favorable clinicopathological features such as lower stage, lower grade, less adjacent carcinoma in situ, and lower Ki-67 proliferation index as well as with better outcome.

H. Yang et al. investigated the correlation between the urine soluble Fas (sFas) and vascular endothelial growth factor (VEGF) in patients with urothelial bladder carcinoma. They reported that the urinary sFas levels and the VEGF expression were correlated significantly and they might play important roles in the occurrence and progression of urothelial bladder cancer.

In the case-control study, S. Choi et al. assessed urinary apurinic/apyrimidine endonuclease 1/redox factor-1 (APE1/Ref-1) by enzyme-linked immunosorbent assay (ELISA) in 169 bladder cancer patients and 108 nonbladder cancer controls. APE1/Ref-1 levels were significantly elevated in bladder cancer patients compared to those in controls and were correlated with tumor grade and stage. These findings suggest that urinary APE1/Ref-1 levels would be clinically applicable for diagnosis of bladder cancer.

Plasmacytoid urothelial carcinoma is a rare and aggressive variant form and little is known about HER2 protein expression and gene alterations. B. Kim et al. demonstrated that HER2 protein overexpression was frequently found and suggested that HER2 may be a potential therapeutic target for this variant form.

The available omics data may allow us to elucidate the mechanisms behind bladder carcinogenesis. According to the omics data on human cells, C.-W. Li and B.-S. Chen constructed an integrated genetic and epigenetic network system (IGEN) based on three coupling regression models. They demonstrate that an accurate genome-wide IGEN would allow us not only to elucidate bladder carcinogenesis mechanisms, but also to improve drug safety and efficacy in the treatment of bladder cancer.

The review by Y. Miyata and H. Sakai demonstrated the various histological and molecular markers for recurrence after intravesical therapy in patients with nonmuscle-invasive bladder cancer. Because intravesical therapy is usually performed after transurethral resection, they discussed the results obtained from tissue samples regarding the various cancer-related molecules, immunity-related factors, and gene polymorphism.

M. Nagata et al. reported the review article discussing the application of molecular predictive biomarkers in patients with advanced muscle-invasive bladder cancer as well as in postcystectomy patients. They also discuss the current findings of liquid biopsy in patients with advanced bladder cancer.

In this special issue, H. S. Kim and J. H. Ku reviewed the clinical studies dealing with systemic inflammatory response (SIR) related biomarkers, with a special focus on neutrophil-to-lymphocyte ratio (NLR). Elevated NLR has shown a significant association with adverse outcomes in patients with carcinoma of upper urinary tract as well as bladder. Since NLR may be an inexpensive and reproducible measurement, it might become a promising tool in the management of urothelial carcinoma.

In one article of this issue, X. Gan et al. conducted a systematic review and meta-analysis and identified an important link between downregulated p16 expression and poor prognosis in patients with bladder cancer. They concluded that p16 plays an essential role in deterioration of bladder cancer and could serve as a biomarker for patients' prognosis.

Taken together, we believe that this special issue gives a comprehensive overview of the contemporary area of diagnostic and prognostic markers in bladder cancer. We hope this special issue is useful to researchers and clinicians to research of bladder cancer.

